# Supplementary material for: The Botrytis cinerea Crh1 transglycosylase is a cytoplasmic effector triggering plant cell death and defense response
Source: Nat Commun. 2021 Apr 12;12:2166. doi: 10.1038/s41467-021-22436-1 (PMC8042016; doi:10.1038/s41467-021-22436-1)
Supplement: Supplementary file 1 — Supplementary Information [file 41467_2021_22436_MOESM1_ESM.pdf]

The *Botrytis cinerea* Crh1 transglycosylase is a cytoplasmic effector triggering plant cell death and defense response

Kai Bi *et, al*

Supplementary Figures

Supplementary Fig. 1. Phylogenetic and multiple sequence alignment of *B. cinerea* Crh family proteins. a Schematic presentation of predicted signal peptide (green) and GH16 domain (blue) of BcCrh proteins. Multiple sequence alignment (MSA) analysis of *B. cinerea* Crh proteins and their homologues from other species was generated by Clustal Omega online tool. Due to the C-terminus residues with low sequence identity, amino acid residues 1-277 of BcCrh1 were used for alignment. **b** Neighbor-joining tree of Crh proteins was generated based on the MSA result. The four *B. cinerea* proteins are marked by stars. **c** MSA results reformatted by Mview. Two highly conserved Cys residues are marked by red stars. Other conserved residues are color shaded. The conserved catalytic activity motif and the acceptor sugar binding motif are boxed by red rectangle. Af – *Aspergillus fumigatus*, Sc – *Sccharomyces cerevisiae*, Ca – *Candida albincas.*

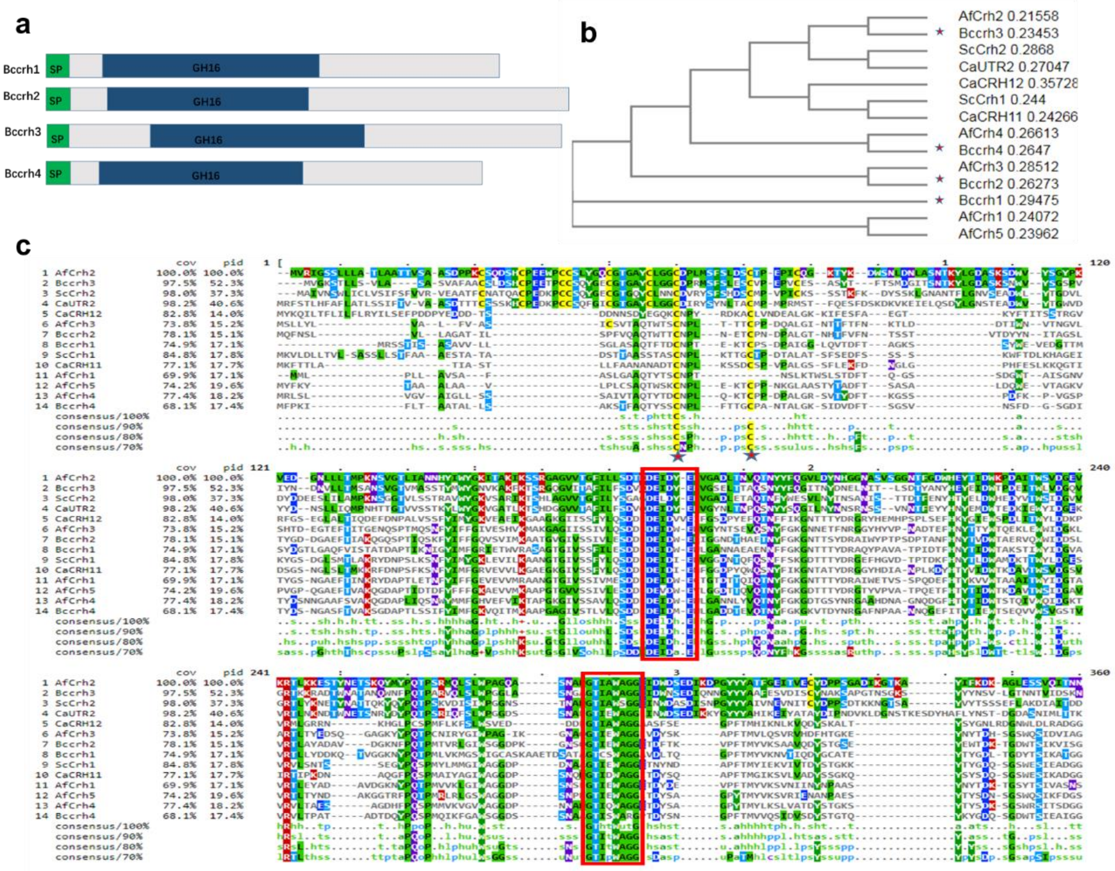

Supplementary Fig. 2. 3D structural models prediction of BcCrh1. HHpred was used for modeling template search against the PDB database. The Crh5 transglycosylase from *Aspergillus fumigatus* (PDB entry 6IBW, UniProt entry Q8J0P4) was identified as the best template for modelling with 48% sequence identity to BcCrh1 (Probability 100%, E-value 1.1e-32). The ConSurf webserver was used to collect homologues from the clean UniProt database, the pairwise alignment of B1084 (later called BcCrh1) and the template was deduced from the multiple sequence alignment. 100 different models were generated by MODELLER-9.18 with default parameters, and following a short energy minimization using GROMACS-5.1 and the AMBER99SB-ILDN force field, the model with the predicted lowest energy was chosen. **a** The model structure of BcCrh1 monomer colored based on evolutionary conservation with turquoise-through-maroon representing variable-through-conserved residues respectively. The C26 and C33 at the N-terminus (highlighted in colored-spheres) form a disulfide bond and are both highly conserved among Crh protein members. **b-g** Predicted potential dimerization interfaces of BcCrh1 colored as in **a**. The PISA webserver (http://www.ebi.ac.uk/pdbe/prot_int/pistart.html) and the crystal structure of 6IBW were used to test potential dimerization interfaces of BcCrh1. Two different topologies of the dimer were predicted based on the analysis. Schematic ribbon diagram of dimerized BcCrh1 model are shown (the top view, bottom view and dimerization interface). **b**, **c** and **f**: "head-to-head" topology of the dimer. **d**, **e** and **g**: "head-to-tail" topology of the dimer.

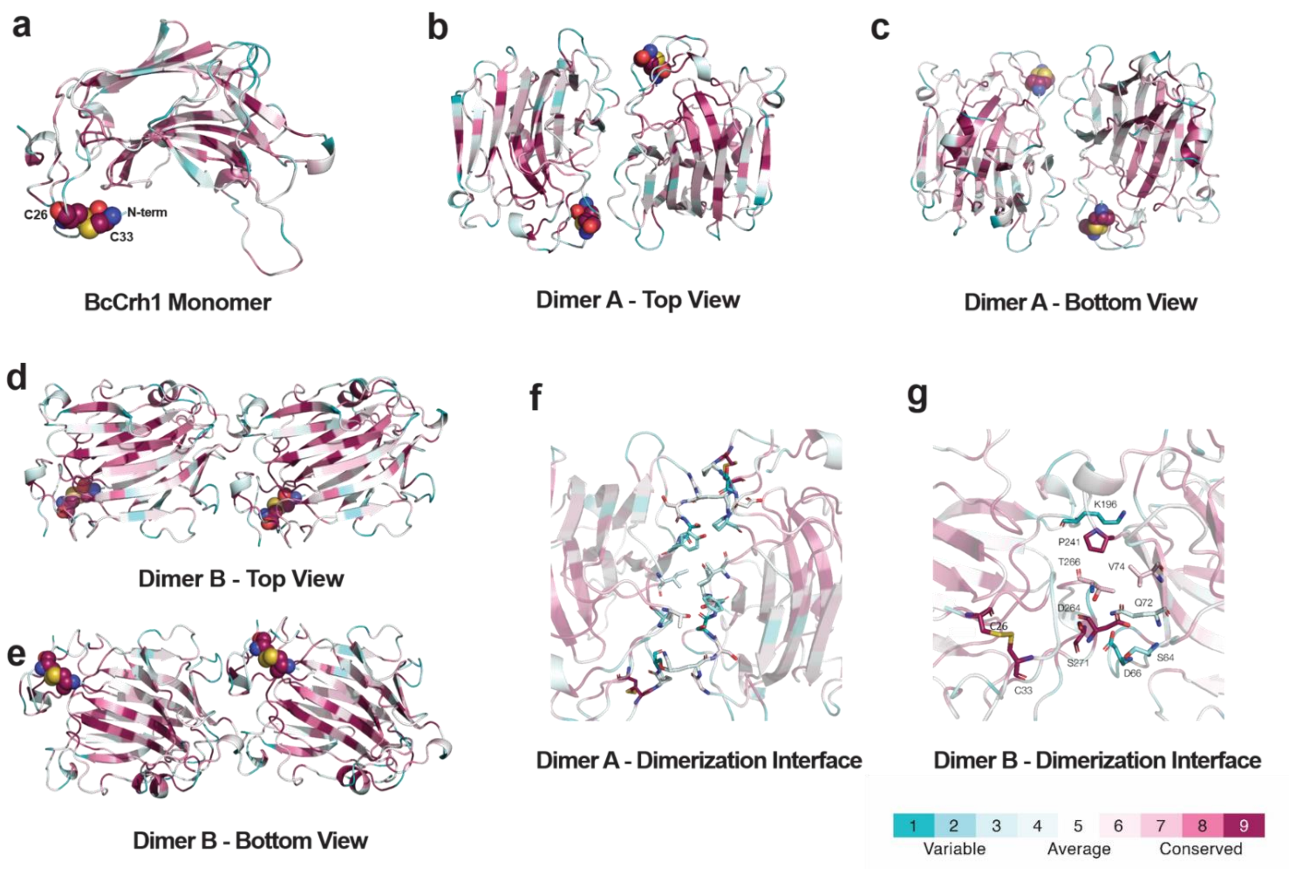

Supplementary Fig. 3. Induction of plant cell death by BcCrh1. **a** *N. benthamiana* leaves were infiltrated with Agrobacterium harbors construct encoding GFP (35S:GFP), native (35S:BcCrh1) or the enzymatic inactive (35S:MBcCrh1) BcCrh1. Images were taken five days after agroinfiltration. **b** Cell death inducing activity of the BcCrh1^{C26AC33A} mutant protein was tested by agroinfiltration assay of *N. benthamiana* leaves. Representative pictures of *N. benthamiana* were taken at 5 d after infiltration. **c** Representative *N. benthamiana* leaves five days after agroinfiltration with constructs expressing BcCrh1$^{21-391}$, CBL1n-BcCrh1$^{21-391}$ (Myristoylation signal), NLS-BcCrh1$^{21-391}$ (Nuclear localization signal) and nls-BcCrh1$^{21-391}$ (Mutant form of nuclear localization signal).

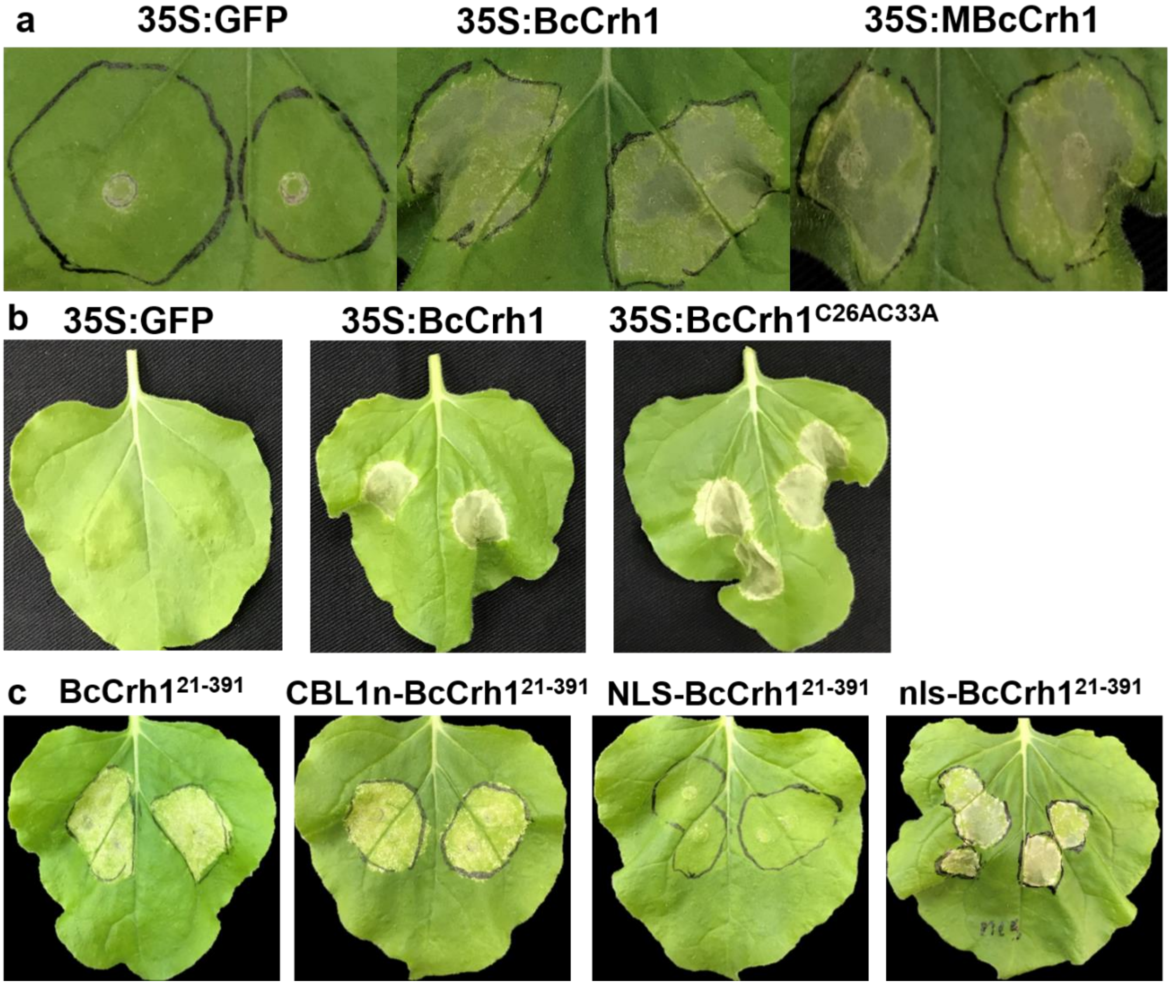

Supplementary Fig. 4. Induction of cell death by BcCrh1 in different plant species. a Response of *N. benthamiana* leaves to different concentrations of purified BcCrh1 and MBcCrh1 protein. Pictures were taken two days after treatment. **b** Infiltration assay of Tomato, *A. thaliana* and maize leaves. Leaves were infiltrated with 11 μM of purified protein, pictures of representative leaves were taken two days after treatment. White dash line shows the boundary of infiltration area.

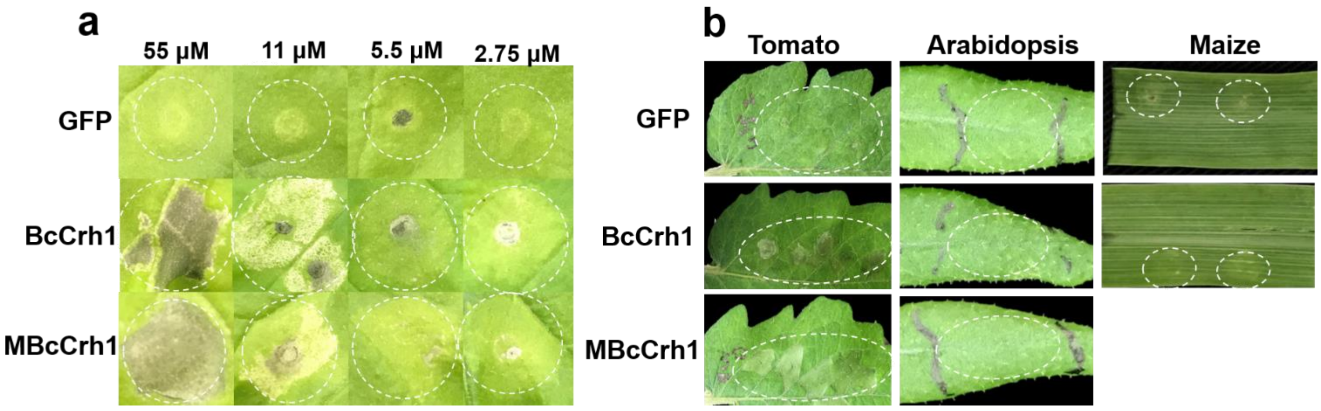

Supplementary Fig. 5. Quantification of necrosis induced by different parts of the BcCrh1 protein. **a** *N. benthamiana* leaves were Agroinfiltrated with the indicated BcCrh1 fragments, leaf samples were collected after five days and stained with trypan blue. **b** Relative staining intensity was measured by ImageJ. For each treatment, 10 samples from three independent biological replications were used for statistical analysis. Center lines of box plots represent the medians, box edges show the 25th and 75th percentiles; whiskers extend to minimum and maximum values from the 25th and 75th percentiles; all present data are indicated as black dots. Different letters indicate statistically significant differences ($P \leq 0.01$, one-way ANOVA).

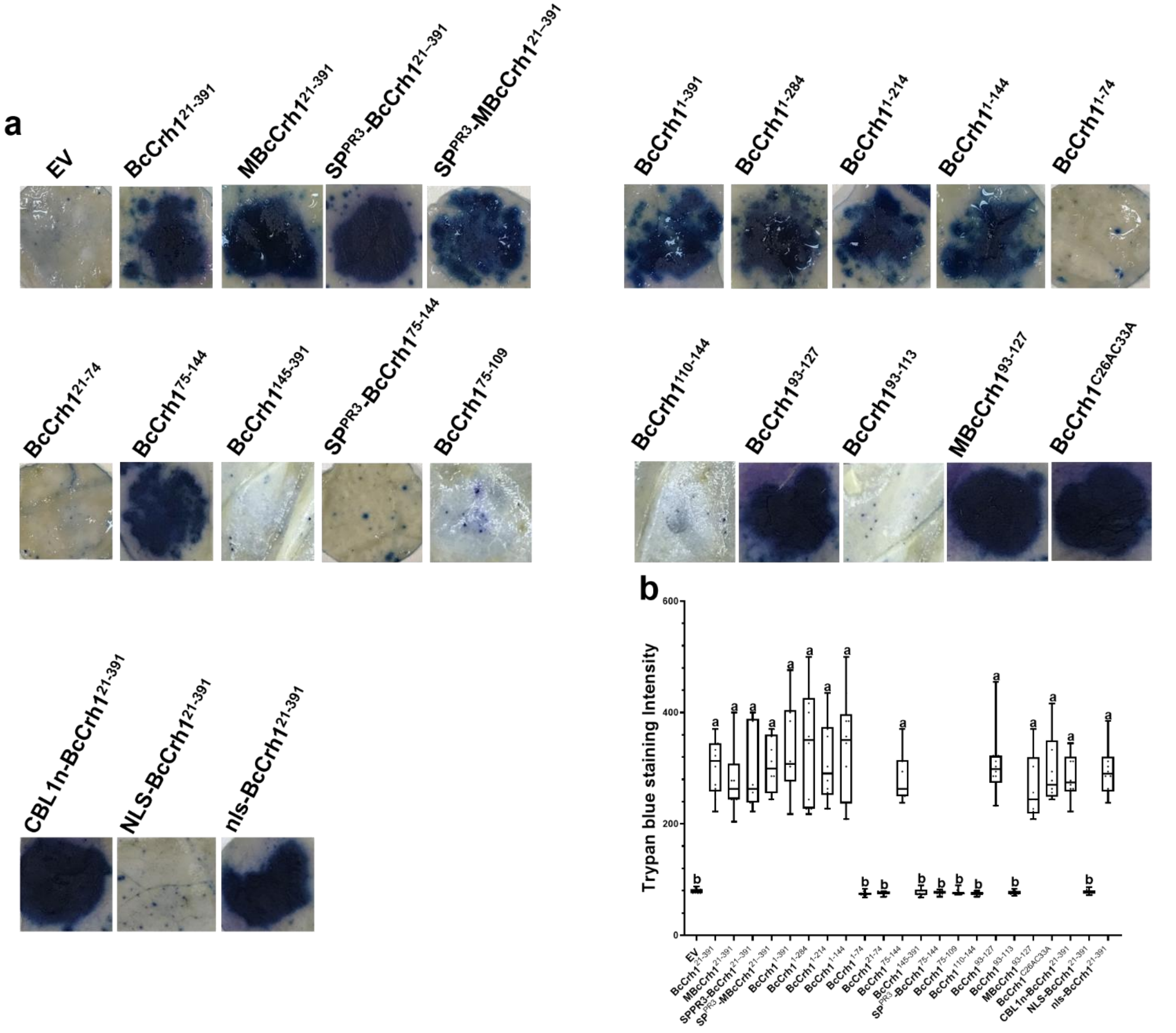

Supplementary Fig. 6. Complementation assay of Crh-deficient *S. cerevisiae* strain. *S. cerevisiae crh1*Δ *crh2*Δ cells, which are completely deficient of Crh activity and hypersensitive to Congo red were transformed with BcCrh1, MBcCrh1 or BcCrh1^{C26AC33A}. **a** Cells of the different strains were grown to mid-log phase at 24°C in SD-Ura overnight, cultures were diluted to 3×10^6 cells ml^{-1} and then further five 1:5 serial dilutions. Five microliters of cell suspension were spotted onto YPD solid medium (control) or YPD supplemented with 50 µg/mL, 100 µg/mL, 150 µg/mL and 200 µg/mL Congo red and then plates were incubated for three days at 30°C. **b** Protein expression. Total proteins were extracts from exponential cultures and subjected to 10% SDS/PAGE and Western blot analysis using anti-c-myc monoclonal antibody (top). Total protein loading was evaluated by coomassie brilliant blue staining (Bottom).

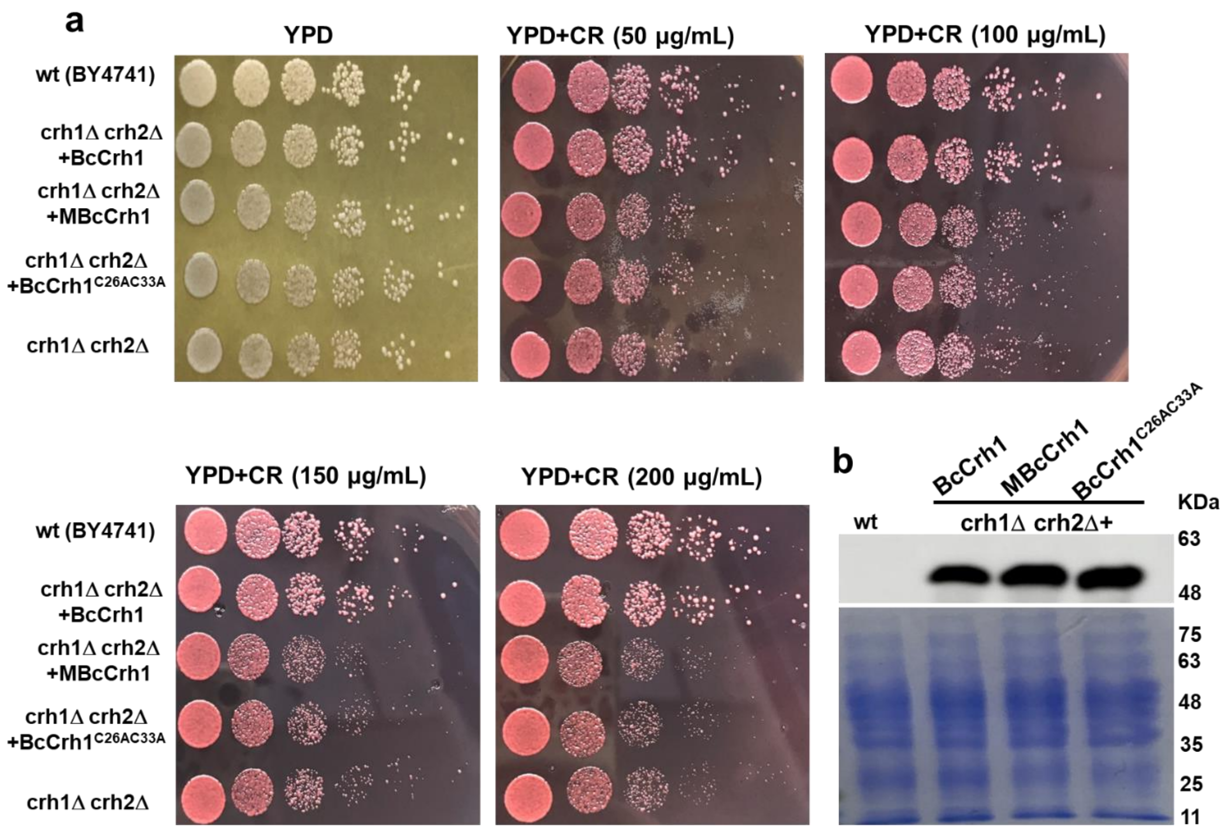

Supplementary Fig. 7. *bccrh1* gene expression during saprophytic and pathogenic development. *bccrh1* expression levels were evaluated by qRT-PCR with the *bcgpdh* as a reference gene for normalization. Mean and standard deviations were calculated from three biological replications. The relative expression levels were calculated using the comparative Ct method. Expression level of *bccrh1* at 0 h was set as 1.

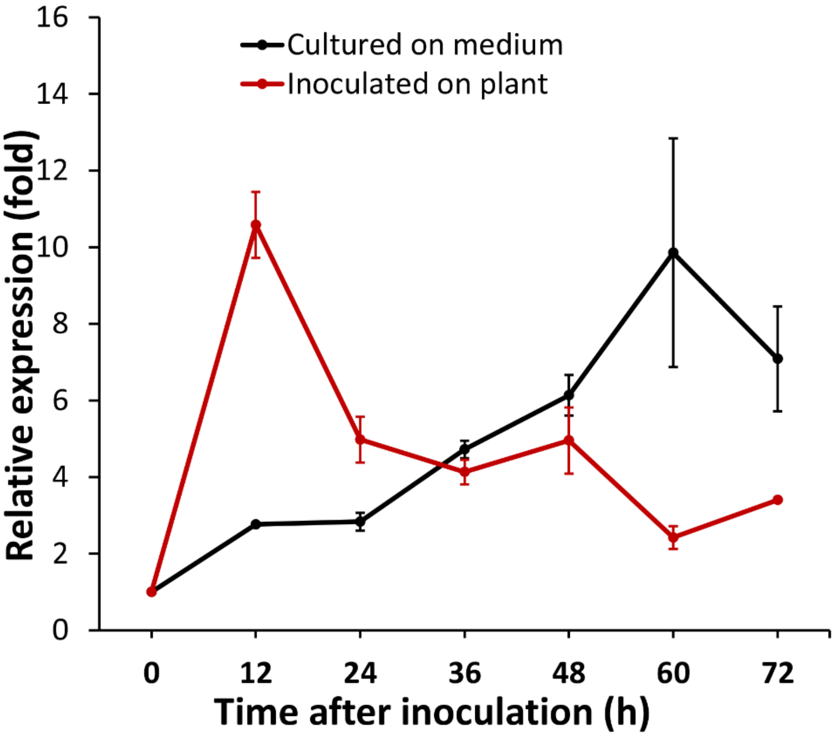

Supplementary Fig. 8. Secretion of Bccrh1 during plant infection. Onion epidermis was inoculated with spore suspension of a *B. cinerea* strain that expresses BcCrh1-GFP fusion protein. Samples were scanned by a confocal microscope. Images show continuous z-series of different infection states. **a, b** Protein localization to hyphal tips in early rime points (12, 21 hpi, arrowhead); **c** Protein localization secretion in infection structures (36 hpi, asterisks). Bar = 20 μm. The experiments were repeated three times with similar results obtained.

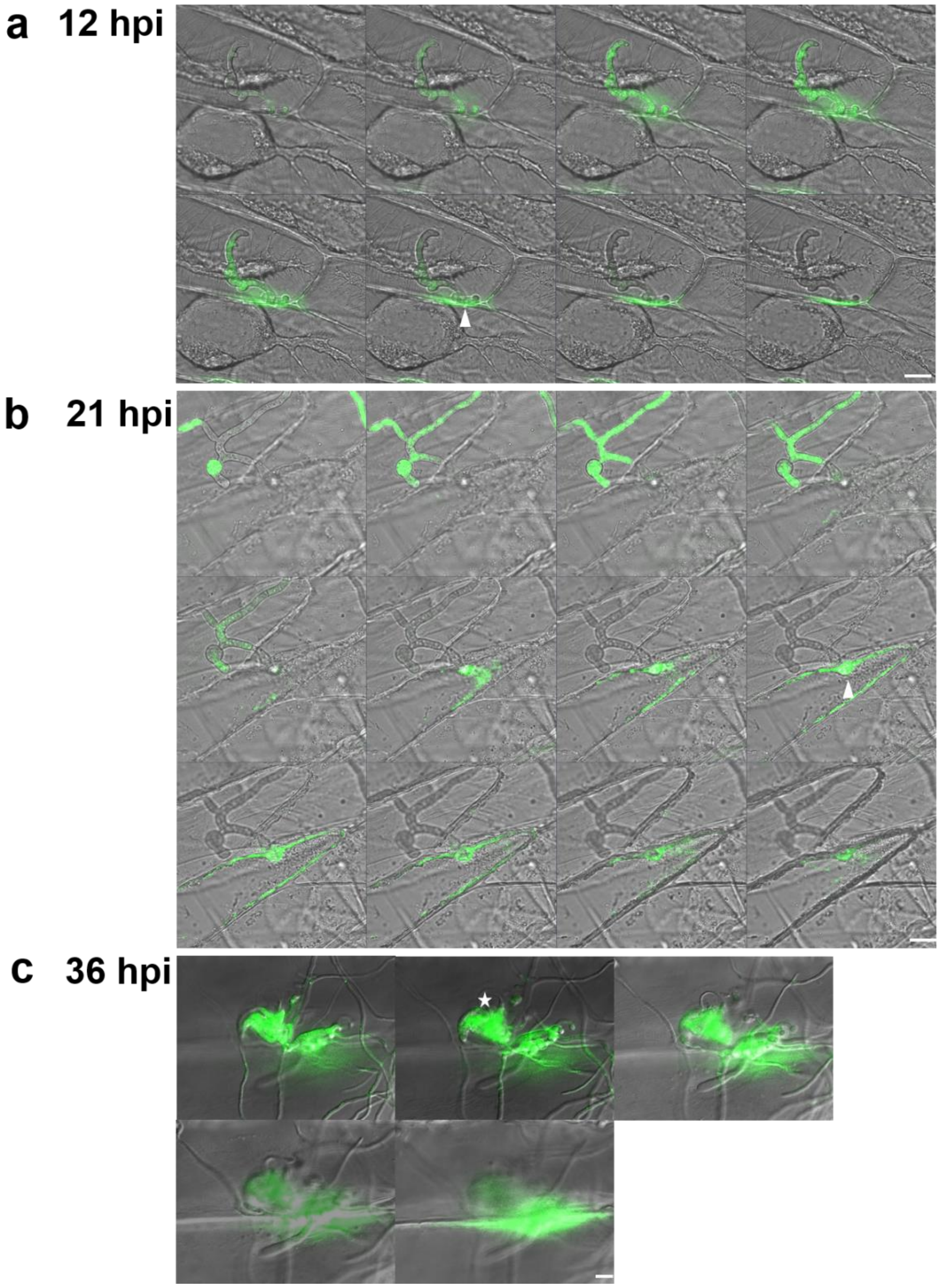

Supplementary Fig. 9. Developmental phenotypes of *B. cinerea* mutant strains. a Hyphal growth rate. Fungi were grown on GB5+Glucose plates at 22°C with continuous fluorescent light. Radial growth was measured daily for three days and growth rate (mm/d) was calculated. Data represent mean ± SD (n = 6) from three independent biological replications. **b** Spore germination. Values stand for mean ± SD from three independent biological replications, at least 120 spores were counted in each replication. **c** Effect of cell wall stressor on colony radial growth. Fungi were cultured on GB5+Glucose medium with or without 300 µg/ml Congo red (CR), colony diameter was measured after three days. Data represent the means ± SD from three independent biological replications. In the graphs, ns = no statistical differences at $P \leq 0.01$ according to one-way ANOVA. **d** Infection cushion formation. Conidia were suspended in GB5+2% Gluc medium, density was adjusted 10^4 conidia ml^{-1} and 10 µl of the suspension were applied to a glass slide and incubated in a moist chamber at 22°C. Pictures were taken after 36 h of incubation. Red rings and arrow indicate the typical and abnormal infection cushions, respectively. Notably, at this stage, all strains except Δ/OE-MBcCrh1 formed normal infection cushions. Strain designation: wt – B05.10 wild type, ΔBcCrh1 – *bccrh1* deletion, OE-BcCrh1 – *bccrh1* over-expression, OE-MBcCrh1 – over-expression of enzyme inactive *bccrh1*, Δ/NP-MBcCrh1 – expression of the enzyme inactive *bccrh1* from native *bccrh1* promoter in background of *bccrh1* deletion, Δ/OE-MBcCrh1^{C26AC33A} – over-expression of enzyme inactive *bccrh1* with mutations in C26 and C33 in background of *bccrh1* deletion. Bar = 20 µm. Images are from a single experiment that was repeated three times with similar results. **e** Pathogenicity assay. Intact and artificially wounded bean leaves were inoculated with wild type and Δ/OE-MBcCrh1 spore suspensions, the plants were incubated in a moist chamber for three days and then pictures were taken and lesion diameter was measured. At least 18 sample points from three independent biological replications were used for statistical analysis. Box limits shows the 25th and 75th percentiles, the center lines of Boxplots indicate the medians values; whiskers extend to minimum and maximum values; all present data are indicated as black dots. Different letters indicate statistical differences at $P \leq 0.01$ according to one-way ANOVA.

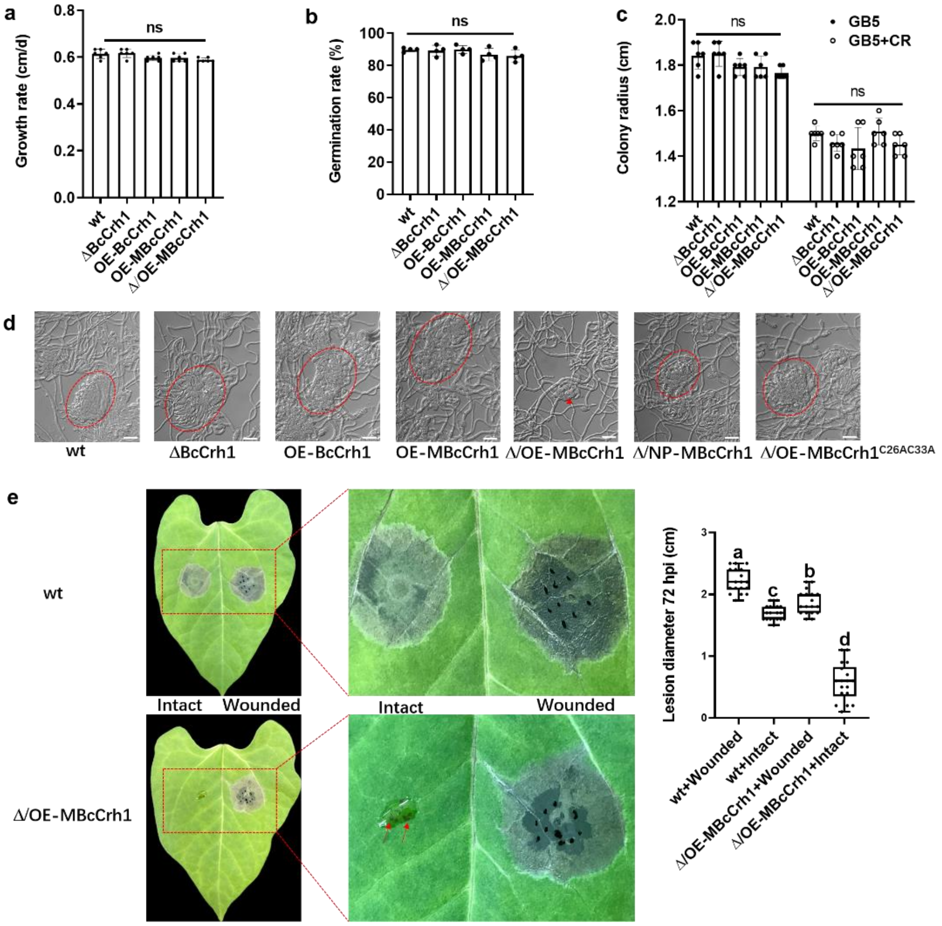

Supplementary Fig. 10. PCR validation of *B. cinerea* mutant strains. **a** Analysis of *bccrh1* deletion strains ΔBcCrh1-2 and ΔBcCrh1-9. **b**-**c** RT-PCR analysis of *bccrh1* expression in different over-expression strains. The *bcactin* gene was used as normalizer. **d** Confirmation of BiFC expression cassettes. Genomic DNA was extracted from the different strains (N'GFP-BcCrh1/C'GFP-BcCrh1, N'GFP-BcCrh1^{C26AC33A}/C'GFP-BcCrh1^{C26AC33A} and N'GFP/C'GFP-BcCrh1) and presence of the BiFC cassette was verified by PCR. NTC – no template control, ddH$_2$O was used as negative control. Images are from a single experiment that was repeated twice with similar results.

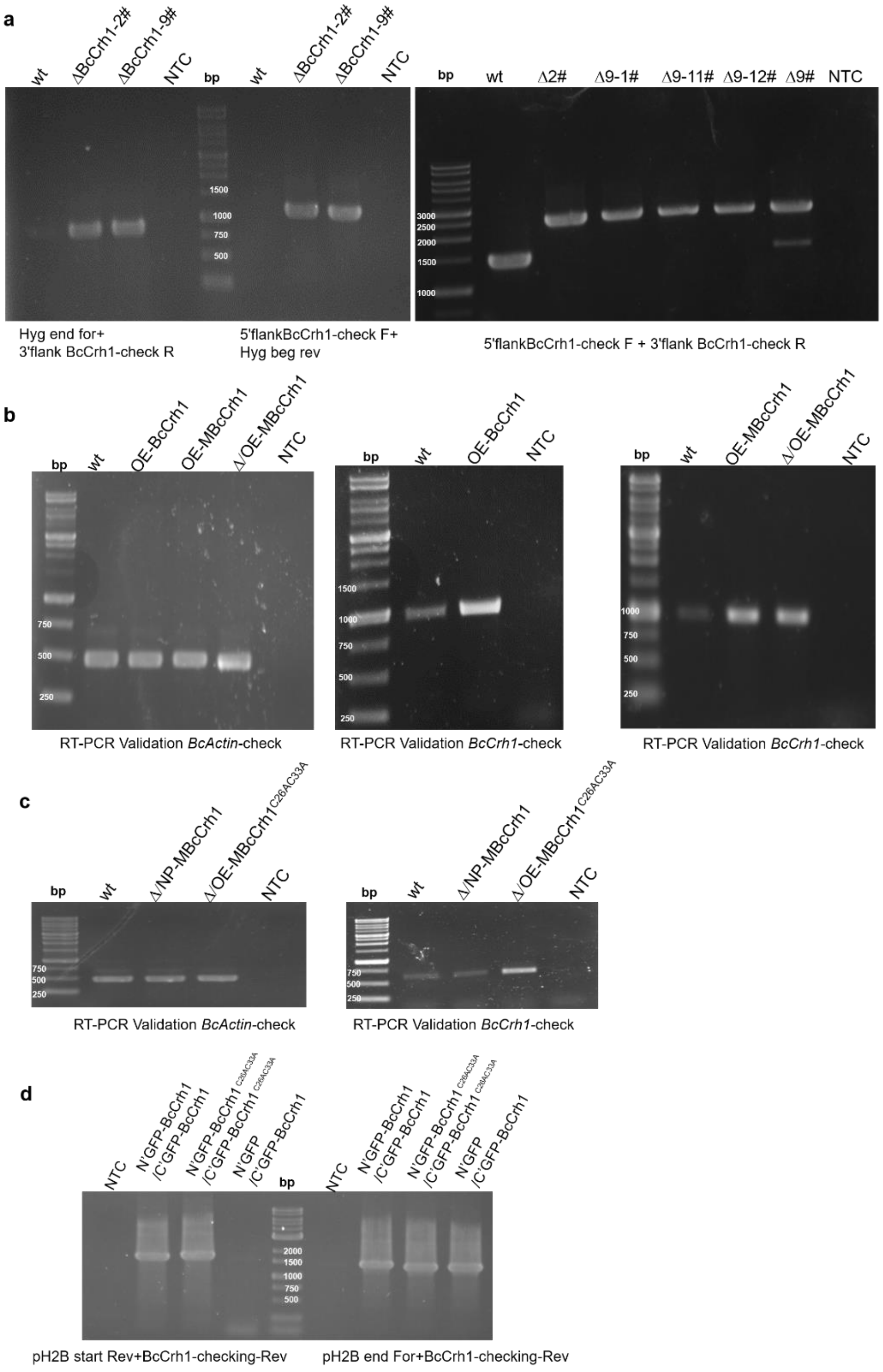

Supplementary Fig. 11. Expression of recombinant proteins. *α*-GFP antibody and *α*-His antibody were used to detect protein expression by Western blot analysis. **a1-a5** Protein fused with GFP tag that were extracted from *N. benthamiana* leaves following Agroinfilration. **b** Proteins isolated from *A. thaliana* transgenic lines expressing the BcCrh1-GFP protein (Line1 and Line2) and the empty vector (EV). **c1** Proteins fused with GFP tag that were produced in *E. coli*. **c2** Coomassie blue staining and Western blots with α-His antibody of proteins with His tag that were produced in *E. coli.* Black arrows indicate the expected products with nonspecific bands. Images are from a single experiment that was repeated twice with similar results.

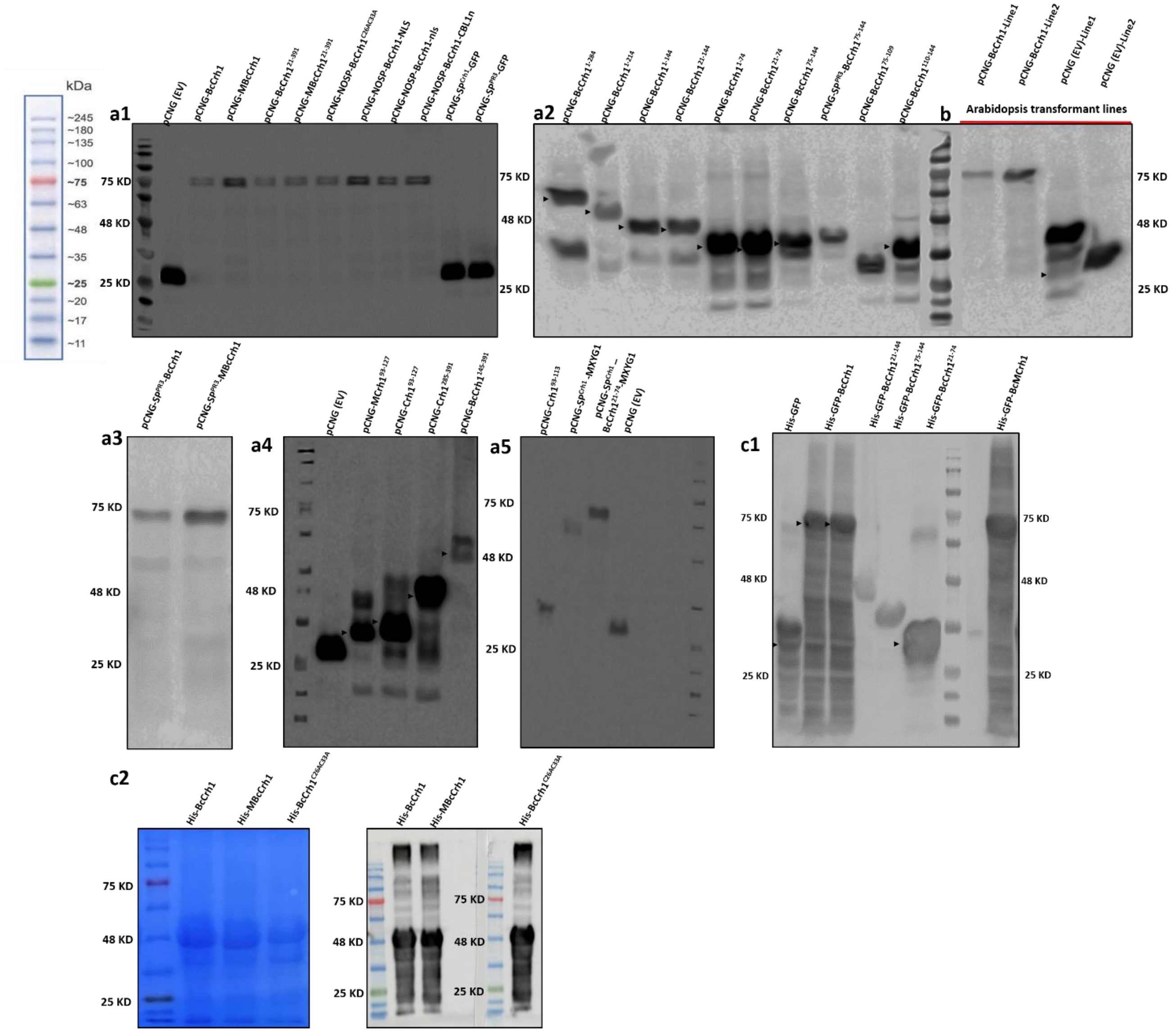

Supplementary Tables

Supplemental Table 1. List of Oligonucleotides used in this study.

Primer purpose	Primer name and sequence (5'- 3')
BcCrh1 transient expression with native SP	*BcCrh1*-TE F: ccatttacgaacgatagcatctagaATGCGTTCCTCAACAATATC
	BcCrh1-TE R: cccgggggtaccgtcgacggatccCATAACGAGATAACCTAATCC
BcCrh1 transient expression without SP	*BcCrh1*$^{21-391}$-TE F: ccatttacgaacgatagcatctagaATGCAAACATTCACTGATTGCAA
	BcCrh1$^{21-391}$-TE R: cccgggggtaccgtcgacggatccCATAACGAGATAACCTAATCC
BcCrh1 transient expression with plant SP (PR3)	*SP(PR3)-BcCrh1*$^{21-391}$-TE F: ccatttacgaacgatagcatctagaATGAAGACTAATCTTTTTCTCT
	SP(PR3)-BcCrh1$^{21-391}$-TE R: cccgggggtaccgtcgacggatccCATAACGAGATAACCTAATCC
BcCrh1$^{1-284}$ transient expression	*BcCrh1*$^{1-284}$-TE F: ccatttacgaacgatagcatctagaATGCGTTCCTCAACAATATC
	BcCrh1$^{1-284}$-TE R: ctcccccgggggtaccgtcgacggatccAGCTGATCCGTCGGCGCTGCA
BcCrh1$^{1-214}$ transient expression	*BcCrh1*$^{1-214}$-TE F: ccatttacgaacgatagcatctagaATGCGTTCCTCAACAATATC
	BcCrh1$^{1-214}$-TE R: ctcccccgggggtaccgtcgacggatccGGCACATCCGATCCAGCTTC
BcCrh1$^{1-144}$ transient expression	*BcCrh1*$^{1-144}$-TE F: ccatttacgaacgatagcatctagaATGCGTTCCTCAACAATATC
	BcCrh1$^{1-144}$-TE R: ctcccccgggggtaccgtcgacggatccCGTCGTGTTTCCCTTTCCGA
BcCrh1$^{1-74}$ transient expression	*BcCrh1*$^{1-74}$-TE F: ccatttacgaacgatagcatctagaATGCGTTCCTCAACAATATC
	BcCrh1$^{1-74}$-TE R: ctcccccgggggtaccgtcgacggatccGACGAATTGGGCACCGAGG
BcCrh1$^{21-74}$ transient expression	*BcCrh1*$^{21-74}$-TE F: ccatttacgaacgatagcatctagaATGCAAACATTCACTGATTGCAA
	BcCrh1$^{21-74}$-TE R: ctcccccgggggtaccgtcgacggatccGACGAATTGGGCACCGAGG
BcCrh1$^{75-144}$ transient expression	*BcCrh1*$^{75-144}$-TE F: ccatttacgaacgatagcatctATGATTAGCACAGCAACCGATGCAC
	BcCrh1$^{75-144}$-TE R: ctcccccgggggtaccgtcgacggatccCGTCGTGTTTCCCTTTCCGA
BcCrh1$^{145-391}$ transient expression	*BcCrh1*$^{75-391}$-TE F: ccatttacgaacgatagcatctATGACCTATGACCGTGCTCAATAC

	BcCrh1$^{75-391}$-TE R:<br>cccgggggtaccgtcgacggatccCATAACGAGATAACCTAATCC
BcCrh1$^{75-144}$ transient expression with plant SP (PR3)	SP (PR3) *BcCrh1*$^{75-144}$-TE F:<br>ccatttacgaacgatagcatctagaATGAAGACTAATCTTTTTCTCT<br>*BcCrh1*$^{75-144}$-TE R:<br>ctcccccgggggtaccgtcgacggatccCGTCGTGTTTCCCTTTCCGA
BcCrh1$^{75-109}$ transient expression	*BcCrh1*$^{75-109}$-TE F:<br>ccatttacgaacgatagcatctATGATTAGCACAGCAACCGATGCAC<br>*BcCrh1*$^{75-109}$-TE R:<br>ctcccccgggggtaccgtcgacggaACTGACGATACCAGTTCCG
BcCrh1$^{110-144}$ transient expression	*BcCrh1*$^{110-144}$-TE F:<br>ccatttacgaacgatagcatctATGAGTTTTATTCTCGAATCCGA<br>*BcCrh1*$^{110-144}$-TE R:<br>ctcccccgggggtaccgtcgacggatccCGTCGTGTTTCCCTTTCCGA
BcCrh1$^{93-127}$ transient expression	*BcCrh1*$^{92-127}$-TE F:<br>ccatttacgaacgatagcatctATGGGTCGCATCGAGACATGGGTG<br>*BcCrh1*$^{92-127}$-TE R:<br>ctcccccgggggtaccgtcgacggaACCGAGCCATTCCCAATCAA
MBcCrh1$^{93-127}$ transient expression	*MBcCrh1*$^{92-127}$-TE F:<br>ccatttacgaacgatagcatctATGGGTCGCATCGAGACATGGGTG<br>*MBcCrh1*$^{92-127}$-TE R:<br>ctcccccgggggtaccgtcgacggaACCGAGCCATTGCCAATGAA<br>TCTGATCGAG
BcCrh1$^{93-113}$ transient expression	*BcCrh1*$^{92-113}$-TE F:<br>ccatttacgaacgatagcatctATGGGTCGCATCGAGACATGGGTG<br>*BcCrh1*$^{92-113}$-TE R:<br>ctcccccgggggtaccgtcgacggaGAGAATAAAACTACTGACG
SP(BcCrh1)-GFP transient expression 35S:*SP(BcCrh1) cloning*	*SP(BcCrh1)-GFP*-TE F:<br>TAAAACGACGGCCAGTGCCAAGCTTAGCTTGCATGCCTGCAGG<br>*SP(BcCrh1)-GFP*-TE F:<br>CCCGCCTCCCCCGGGGGTACCTGCACTGGCAAGTCCTGAAAG
BcCrh1^{C26AC33A} transient expression	*BcCrh1*^{C26AC33A}-TE F:<br>CCATTTACGAACGATAGCATCTAGAATGCAAACATTCACTGAT<br>GCTAAC<br>*BcCrh1*^{C26AC33A}-TE R:<br>cccgggggtaccgtcgacggatccCATAACGAGATAACCTAATCC
BcXYG1 Fused with *SP(BcCrh1)* and *GFP* transient expression	*SP(BcCrh1)- BcXYG1* F:<br>ATGCGTTCCTCAACAATATCAGCATCTGCTGTTGTTCTCCTTTC<br>AGGACTTGCCAGTGCAAACCCTACTCCTACTCTTG<br>*BcXYG1-GFP* R:<br>ctcccccgggggtaccgtcgacggatccATTGAGCGAGACGGAGTAG
BcXYG1 Fused with *SP(BcCrh1)*-*BcCrh1*$^{21-74}$ and *GFP* transient	*SP(BcCrh1)- BcCrh1*$^{21-74}$ F:<br>ccatttacgaacgatagcatctagaATGCGTTCCTCAACAATATC

expression	*SP(BcCrh1)- BcCrh1*$^{21\text{-}74}$ R:<br>GACGAATTGGGCACCGAGG<br>*BcCrh1-BcXYG1* F:<br>CCTCGGTGCCCAATTCGTCAACCCTACTCCTACTCTTG<br>*BcCrh1-BcXYG1* R:<br>ctcccccgggggtaccgtcgacggatccATTGAGCGAGACGGAGTAG
BcCrh1 over-expression	*BcCrh1*-OE F:<br>attggcgcgccATGCGTTCCTCAACAATATCAGC<br>*BcCrh1*-OE R:<br>gcggccgcTTAAGCGTAATCTGGAACATCGTATGGGTACATAACG<br>AGATAACCTAATCCGAGAC
MBcCrh1 over-expression in wt and *bccrh1* deletion mutant background<br>*MBcCrh1*^{C26AC33A} over-expression in *bccrh1* deletion mutant background	*MBcCrh1-OE (PoliC-MBcCrh1)* F:<br>CCATCACATCACAATCGATCCAACCATGCGTTCCTCAACAATA<br>TCAGCAT
	MBcCrh1-OE (*MBcCrh1-Tgluc*) R:<br>CATACATCTTATCTACATACGCTAAGCGGCCGCCATAACGAGA<br>TAACCTAATCCGAGA
BcCrh1-GFP fused protein under control of the native *bccrh1* promoter	*PtrpC-promoter of BcCrh1* F:<br>GCCCAAAAAATGCTCCTTCAATATCACTAGTTTCATATGCTTTG<br>GAAATGGGATGG<br>*Promoter of BcCrh1* R:<br>GCTGATATTGTTGAGGAACGCATCTTGAAATATGTGATGTGTG<br>TGTGC<br>*BcCrh1* F:<br>ATGCGTTCCTCAACAATATCAGCAT<br>*BcCrh1-GFP*-R:<br>TACTTACCTCACCCTTGGAAACCATCATAACGAGATAACCTAA<br>TCCGAGA
MBcCrh1 under control of the native promoter in *bccrh1*deletion mutant background	*PtrpC-promoter of BcCrh1* F:<br>GCCCAAAAAATGCTCCTTCAATATCACTAGTTTCATATGCTTTG<br>GAAATGGGATGG<br>*promoter of BcCrh1* R:<br>GCTGATATTGTTGAGGAACGCATCTTGAAATATGTGATGTGTG<br>TGTGC<br>*MBcCrh1* F:<br>ATGCGTTCCTCAACAATATCAGCAT<br>*MBcCrh1-Tgluc-R*:<br>CATACATCTTATCTACATACGCTAAGCGGCCGCCATAACGAGA<br>TAACCTAATCCGAGA
BcCrh1/MBcCrh1 expression in *E. coli*	*BcCrh1* pET14b F:<br>AACCTGTACTTCCAGGGTCATATGCAAACATTCACTGATTG<br>*BcCrh1* pET14b R:

	CCGGCCATGGAAAAAAAACATTACATAACGAGATAACCTAATC
BcCrh1$^{21\text{-}144}$ expression in *E. coli*	*BcCrh1*$^{21\text{-}144}$ pET14b F: AACCTGTACTTCCAGGGTCATATGCAAACATTCACTGATTGC<br>*BcCrh1*$^{21\text{-}144}$ pET14b R: TCGGGCTTTGTTAGCAGCCGTTACGTCGTGTTTCCCTTTCCGA
BcCrh1$^{75\text{-}144}$ expression in *E. coli*	*BcCrh1*$^{75\text{-}144}$ pET14b F: AACCTGTACTTCCAGGGTCATATGATTAGCACAGCAACCGATGCAC<br>*BcCrh1*$^{75\text{-}144}$ pET14b R: TCGGGCTTTGTTAGCAGCCGTTACGTCGTGTTTCCCTTTCCGA
Generate pET14b linearized vector skeleton for cloning	pET14b-GFP F: CGGCTGCTAACAAAGCCCGAAAGG<br>pET14b-GFP R: CATATGACCCTGGAAGTACAGG
BcCrh1 replacement vector upstream Amplify 5’flank of *BcCrh1*	pTZ-5'flank*BcCrh1* F: ggtaacgccagggttttcccagtcacgacgCCCCCGCCCCTCGTTTGCTTACAGTGAC<br>HygTer-5'flank*BcCrh1* R: cctttttttttcaggaattattctcacagtCTTGAAATATGTGATGTGTGTGTGCGTG
BcCrh1 replacement vector downstream Amplify 3’flank of *BcCrh1*	OliCProm-3'flank*BcCrh1* F: gatctagatgcattcgcgaggtaccgagctACCTGATAAACGAGAAATTCCAGGAAAG<br>pTZ-3'flank*BcCrh1* R: agcggataacaatttcacacaggaaacagcCATGGGAGTATTGCATCAGGTATCACC
Amplify *hph* cassette	OliCprom-Hyg-pOnex F: AGCTCGGTACCTCGCGAATGC<br>ToACR R: ACTGTGAGAATAATTCCTG
Check *BcCrh1* deletion	5'flank*BcCrh1*-check F: TTTCATGACAATGCTGAAAACCTTC<br>3'flank*BcCrh1*-check R: GAGATGTTGAGAATCTAATTACTATT
Used to check homologous recombination of *hph*-containing cassette	Hyg end for: ATTCCCAATACGAGGTCGCCAACATCTTCTTC<br>Hyg beg rev: GAGATGCAATAGGTCAGGCTCTCGCTGAATTC
Used to check the expression cassettes of pH2B: *N’GFP-BcCrh1* and pH2B: *C’GFP-BcCrh1*	pH2B start Rev: caaaacatctctcctccatccacccaaaac<br>pH2B end For: actcatcaacatcaatctcagcc
To amplify part of the open reading frame	*BcCrh1*-check For:

of *BcCrh1*	AGAAAAGACCTGTCCTTCAGACCCA
	BcCrh1-check Rev: AAGATCAATCATGCCGTATTCGTGC
To amplify part of the open reading frame of *BcActin*	*BcActin*-check F: CCCAATCAACCCAAAGTCCAACAG
	BcActin-check R: CCACCGCTCTCAAGACCCAAGA
Y2H assay	*AD-BcCrh1* F: tatggccatggaggccagtgaattcCAAACATTCACTGATTGC
	AD-BcCrh1 R: tctgcagctcgagctcgatgTTACATAACGAGATAACCTAATC
	BD-BcCrh1 F: GCATATGGCCATGGAGGCCGAATTCCAAACATTCACTGATTGC
	BD-BcCrh1 R: GCGGCCGCTGCAGGTCGACGTTACATAACGAGATAACCTAATC
	BD-BcCrh1 ^{C26AC33A} F: GCATATGGCCATGGAGGCCCAAACATTCACTGATGCTAACCCAACAGAAAAGACCGCTCCTTCAGACCCAGCCATTG
	BD-BcCrh1- ^{C26A} F: GCATATGGCCATGGAGGCCGAATTCCAAACATTCACTGATGCTAACCCAACAGAAAAGACCGCT
	AD- BcCrh1$^{\Delta21\text{-}38}$ F: TATGGCCATGGAGGCCAGTGAATTCATTGGAGGCCTTCAAGTGACC
	AD- BcCrh1 $^{\Delta21\text{-}38}$ R: GCATATGGCCATGGAGGCCGAATTCATTGGAGGCCTTCAAGTGACC
BcCrh1 for qRT-PCR	*BcCrh1*-q F: TGATTGCAACCCAACAGAAA
	BcCrh1-q R: GGTTGCTGTGCTAATGACGA
B. cinerea Bcgpdh for qRT-PCR	*Bcgpdh*-q F: CGAAGAATAGCACAAACAGCTGGAC
	Bcgpdh-q R: CGTCACCTTATGCTTCTTGCTCC
S. lycopersicum LoxD for qRT-PCR	*LoxD*-q F: GGCTTGCTTTACTCCTGGTC
	LoxD-q R: AAATCAAAGCGCCAGTTCTT
S. lycopersicum PR1a for qRT-PCR	*PR1a*-q F: TGGTATGGCGTAAGTCGGTA
	PR1a-q R: CTTGGAATCAAAGTCCGGTT
S. lycopersicum ACS6 for qRT-PCR	*ACS6*-q F: CCTGGTTGTTCATTTCATTGCTCAGAG
	ACS6-q R: GCAACTTCAACTCCCTTATTTGGTTGTAA
S. lycopersicum LRR22 for qRT-PCR	*LRR22*-q F: AAGATTGGAGGTTGCCATTGGAGC
	LRR22-q R: ATCGCGATGAATGATCGGTGGAGT
S. lycopersicum PI-II for qRT-PCR	*PI-II*-q F: CCTATTCAAGATGTCCCCGTTC
	PI-II-q R: GGGCAATCCAGAAGATGG

S. lycopersicum NPR1 for qRT-PCR	*NPR1*-q F: CCCTGGCTAGCATGAGGAAG
	NPR1-q R: AGAGAGAGCCCTAAGCCGAT
S. lycopersicum OPR3 for qRT-PCR	*OPR3*-q F: CTTTGAGGAACGCGTATCAGG
	OPR3-q R: TGACACGAGATCAGCATCACC
S. lycopersicum actin for qRT-PCR	*actin*-q F: TGAGCTTCGAGTTGCTCCTGA
	actin-q R: AGCACAGCCTGGATAGCAACA
A. thaliana UBQ10 for qRT-PCR	*UBQ10*-q F: AGATCCAGGACAAGGAAGGTATTC
	UBQ10-q R: CGCAGGACCAAGTGAAGAGTAG
A. thaliana NPR1 for qRT-PCR	*NPR1*-q F: TGCATCAGAAGCAACTTTGG
	NPR1-q R: GGCCTTTGAGAGAATGCTTG
A. thaliana PR5 for qRT-PCR	*PR5*-q F: GTCAATTCAAATCCTCCATCG
	PR5-q R: CGCAGGACCAAGTGAAGAGTAG
A. thaliana PAD4 for qRT-PCR	*PAD4*-q F: TCTTCAGTTAAAGATCAAGGAAGG
	PAD4-q R: GGTTGAATGGCCGGTTATC
A. thaliana PDF1.2 for qRT-PCR	*PDF1.2*-q F: GTTCTCTTTGCTGCTTTCGAC
	PDF1.2-q R: GCAAACCCCTGACCATGT
A. thaliana LOX3 for qRT-PCR	*LOX3*-q F: CGCCAATCAACAGTTTCTGA
	LOX3-q R: CTCGTCTCGTGGCACATACA
BcXYG1$^{E120Q/D122H/E124Q}$ enzymatic activity site-directed mutation	BcXYG1$^{E120Q/D122H/E124Q}$ F: CTCGATCAGATTCATTGGCAATGGCT
	BcXYG1$^{E120Q/D122H/E124Q}$ R: AGCCATTGCCAATGAATCTGATCGAG

Supplemental Table 2. List of *B. cinerea* and yeast strains used in this study.

Strain	Genotype and description	Source
B05.10	Haploidized *B. cinerea* wild type strain	Shlezinger et al., (2016)
Δ*bccrh1*	*bccrh1* deletion mutant	This work
OE-BcCrh1	Over expression strain of the native form of BcCrh1	This work
OE-MBcCrh1	Over expression strain of the mutated form of BcCrh1 that has no enzymatic activity in wild-type background	This work
NP-BcCrh1-GFP	BcCrh1-GFP fused protein under control of the native *bccrh1* promoter	This work
Δ/OE-MBcCrh1	Over expression strain of the mutated form of BcCrh1 that has no enzymatic activity in *bccrh1* deletion background	This work
Δ/NP-MBcCrh1	Mutant strain of the enzymatic inactive BcCrh1 protein under control of the native *bccrh1* promoter in *bccrh1* deletion background	This work
Δ/OE-MBcCrh1^{C26AC33A}	Over expression strain of the mutated form of BcCrh1 that lost both enzymatic activity and homodimer activity in *bccrh1* deletion background	This work
N'GFP-BcCrh1 /C'GFP-BcCrh1	BiFC assay, BcCrh1 was fused with N'GFP and C'GFP, respectively. Co-expressing of the N'GFP-BcCrh1 and C'GFP-BcCrh1 was controlled by *B. cinerea* H2B promoter.	This work
N'GFP-BcCrh1^{C26AC33A} /C'GFP-BcCrh1^{C26AC33A}	BiFC assay, BcCrh1^{C26AC33A} was fused with N'GFP and C'GFP, respectively. Co-expressing of the N'GFP-BcCrh1^{C26AC33A} and C'GFP-BcCrh1^{C26AC33A} was controlled by *B. cinerea* H2B promoter.	This work
N'GFP /C'GFP-BcCrh1	BiFC assay, BcCrh1 was fused with C'GFP only. Co-expressing of the N'GFP and C'GFP-BcCrh1 was controlled by *B. cinerea* H2B promoter.	This work
OE-GFP	Over expression strain of the free GFP protein	This work
BY4741	MATa his3Δ1 leu2Δ0 met15Δ0 ura3Δ0	Cabib E et al., (2007)
GRA007 (crh1Δ crh2Δ)	MATa his3Δ1 leu2Δ0 ura3Δ0 crh2::HIS3 crh1::hphMX4	Cabib E et al., (2007)
crh1Δ crh2Δ/BcCrh1	Yeast strain of the BcCrh1 protein under control of *CRH2* promoter and terminator in GRA007 background	This work

crh1Δ crh2Δ/MBcCrh1	Yeast strain of the enzymatic inactive BcCrh1 protein under control of *CRH2* promoter and terminator in GRA007 background	This work
crh1Δ crh2Δ/BcCrh1^{C26AC33A}	Yeast strain of the BcCrh1^{C26AC33A} protein (the mutated form of BcCrh1 that lost homodimer activity) under control of *CRH2* promoter and terminator in GRA007 background	This work
