# Supplementary material for: The Botrytis cinerea Crh1 transglycosylase is a cytoplasmic effector triggering plant cell death and defense response
Source: Nat Commun. 2021 Apr 12;12:2166. doi: 10.1038/s41467-021-22436-1 (PMC8042016; doi:10.1038/s41467-021-22436-1)
Supplement: Supplementary file 3 — Reporting Summary [file 41467_2021_22436_MOESM3_ESM.pdf]

nature research

Corresponding author(s): Amir Sharon

Last updated by author(s): Feb 28, 2021

# Reporting Summary

Nature Research wishes to improve the reproducibility of the work that we publish. This form provides structure for consistency and transparency in reporting. For further information on Nature Research policies, see our Editorial Policies and the Editorial Policy Checklist.

## Statistics

For all statistical analyses, confirm that the following items are present in the figure legend, table legend, main text, or Methods section.

| n/a | Confirmed | |
|---|---|---|
| ☐ | ☒ | The exact sample size (*n*) for each experimental group/condition, given as a discrete number and unit of measurement |
| ☐ | ☒ | A statement on whether measurements were taken from distinct samples or whether the same sample was measured repeatedly |
| ☐ | ☒ | The statistical test(s) used AND whether they are one- or two-sided<br>*Only common tests should be described solely by name; describe more complex techniques in the Methods section.* |
| ☐ | ☒ | A description of all covariates tested |
| ☐ | ☒ | A description of any assumptions or corrections, such as tests of normality and adjustment for multiple comparisons |
| ☐ | ☒ | A full description of the statistical parameters including central tendency (e.g. means) or other basic estimates (e.g. regression coefficient) AND variation (e.g. standard deviation) or associated estimates of uncertainty (e.g. confidence intervals) |
| ☐ | ☒ | For null hypothesis testing, the test statistic (e.g. *F*, *t*, *r*) with confidence intervals, effect sizes, degrees of freedom and *P* value noted<br>*Give P values as exact values whenever suitable.* |
| ☒ | ☐ | For Bayesian analysis, information on the choice of priors and Markov chain Monte Carlo settings |
| ☒ | ☐ | For hierarchical and complex designs, identification of the appropriate level for tests and full reporting of outcomes |
| ☒ | ☐ | Estimates of effect sizes (e.g. Cohen's *d*, Pearson's *r*), indicating how they were calculated |

*Our web collection on statistics for biologists contains articles on many of the points above.*

## Software and code

Policy information about availability of computer code

| | |
|---|---|
| Data collection | Zeiss LSM780 confocal microscope system and ZEISS ZEN 3.0 (blue edition) imaging Software were used for Confocal imaging and data collection. Zeiss Axio imager M1 microscope and Carl Zeiss AxioVision Rel. 4.8 Software were used for Epifluorescence and light microscopy. qRT-PCR data was collected by MxPro qPCR Software (Agilent) and StepOne Software v2.3 (Applied Biosystems). |
| Data analysis | SignalP-5.0 (http://www.cbs.dtu.dk/services/SignalP/) and TMHMM version 2.0 (http://www.cbs.dtu.dk/services/TMHMM/) for sequence analysis. SMART software (http://smart.embl.de/) for conserved protein domain search. Clustal Omega (https://www.ebi.ac.uk/Tools/msa/) for Multiple Sequence Alignment (MSA). MView Version 1.63 was used to present the MSA result. HHpred (https://toolkit.tuebingen.mpg.de/tools/hhpred) for the prediction of the 3D structural models. ConSurf (2016) was used to collect homologues to produce the pairwise alignment between the two proteins. MODELLER 9.20 (https://salilab.org/modeller/) was used to produce different models, and each model underwent a short energy minimization using GROMACS 2018 and the AMBER99SB-ILDN force field (https://www.nvidia.com/es-la/data-center/gpu-accelerated-applications/gromacs/). PISA 2018 (https://www.ebi.ac.uk/pdbe/pisa/) was used to test potential dimerization interfaces. PyMOL 2.4 (https://pymol.org/2/) for visualization of 3D structural models. The Microsoft Excel 2016 and GraphPad Prism 8 were used for statistics and bar graphs overlaid with dot plots. Image J software (version: 1.60) for the quantification of DAB and Trypan blue staining. |

For manuscripts utilizing custom algorithms or software that are central to the research but not yet described in published literature, software must be made available to editors and reviewers. We strongly encourage code deposition in a community repository (e.g. GitHub). See the Nature Research guidelines for submitting code & software for further information.

## Data

Policy information about availability of data

All manuscripts must include a data availability statement. This statement should provide the following information, where applicable:

- Accession codes, unique identifiers, or web links for publicly available datasets
- A list of figures that have associated raw data
- A description of any restrictions on data availability

The data supporting the results of this study are available within the article and its Supplementary Information files. The authors declare that the other data supporting the findings of this study are available from the corresponding author upon request.

# Field-specific reporting

Please select the one below that is the best fit for your research. If you are not sure, read the appropriate sections before making your selection.

[x] Life sciences  [ ] Behavioural & social sciences  [ ] Ecological, evolutionary & environmental sciences

For a reference copy of the document with all sections, see nature.com/documents/nr-reporting-summary-flat.pdf

# Life sciences study design

All studies must disclose on these points even when the disclosure is negative.

| | |
|---|---|
| Sample size | Sample size was not predetermined based on statistical methods, but were used according to common practice and previous studies. For the transformation of B. cinerea and characterization of the transformants, at least three independent single spore isolates from independent colonies were obtained for each strain (according to Ma, Liang, et al. Molecular plant pathology 18.2 (2017): 263-275). We generated at least two independent A. thaliana transgenic lines and and more than three individual plants of each lines to observe the phenotype (according to Liu, Jun, et al. Nature Plants 6.9 (2020): 1106-1115.) |
| Data exclusions | No data were excluded from analyses in the experiments. |
| Replication | At least three independent biological replications for all assays, and noted in the figure legends or methods section. All biological replications confirmed the similar results. |
| Randomization | For all N. benthamiana plants and Agro-bacterial strains, B. cinerea strains and French beans, Arabidopsis transgenic plants, were maintained and grown under similar controlled conditions. All samples were randomly selected throughout this study. |
| Blinding | Blinding is not relevant in our study. Blinding during the research was not necessary because different mutants and transgenic lines were used in the study, and the differences between samples under different conditions were visually apparent. |

# Reporting for specific materials, systems and methods

We require information from authors about some types of materials, experimental systems and methods used in many studies. Here, indicate whether each material, system or method listed is relevant to your study. If you are not sure if a list item applies to your research, read the appropriate section before selecting a response.

### Materials & experimental systems

| n/a | Involved in the study | |
|---|---|---|
| [ ] | [x] | Antibodies |
| [x] | [ ] | Eukaryotic cell lines |
| [x] | [ ] | Palaeontology and archaeology |
| [x] | [ ] | Animals and other organisms |
| [x] | [ ] | Human research participants |
| [x] | [ ] | Clinical data |
| [x] | [ ] | Dual use research of concern |

### Methods

| n/a | Involved in the study | |
|---|---|---|
| [x] | [ ] | ChIP-seq |
| [x] | [ ] | Flow cytometry |
| [x] | [ ] | MRI-based neuroimaging |

## Antibodies

| | |
|---|---|
| Antibodies used | mouse anti-GFP, cat. no. sc-9996, Santa Cruz Biotechnology, 1:5000 dilution in TBST buffer;<br>mouse anti-His, cat. no. H1029, Sigma-Aldrich, 1:5000 dilution in TBST buffer;<br>mouse anti-c-Myc, cat. no. sc-40, Santa Cruz Biotechnology, 1:5000 dilution in TBST buffer;<br>rabbit anti-HA, cat. no. ab9110, Abcam, 1:2500 dilution in TBST buffer;<br>rabbit anti-Myc, cat. no. ab9106, Abcam, 1:2500 dilution in TBST buffer;<br>goat anti-mouse IgG (whole molecule)-peroxidase-conjugate antibody, cat. no. sc-516102, Santa Cruz Biotechnology, 1:10000 |



April 2020

dilution in TBST buffer;
goat anti-rabbit IgG HRP antibody, cat. no. ab205718 Abcam, 1:10000 dilution in TBST buffer;

Validation

Antibodies were validated by the manufacturers and further evaluated using the proper negative controls.
mouse anti-GFP, cat. no. sc-9996, Santa Cruz Biotechnology: https://datasheets.scbt.com/sc-9996.pdf;
mouse anti-His, cat. no. H1029, Sigma-Aldrich: https://www.sigmaaldrich.com/catalog/product/sigma/h1029?lang=en&region=IL;
mouse anti-c-Myc, cat. no. sc-40, Santa Cruz Biotechnology: https://www.scbt.com/p/c-myc-antibody-9e10;
rabbit anti-HA, cat. no. ab9110, Abcam: https://www.abcam.com/ha-tag-antibody-chip-grade-ab9110.html;
rabbit anti-Myc, cat. no. ab9106, Abcam: https://www.abcam.com/myc-tag-antibody-ab9106.html;
goat anti-mouse IgG (whole molecule)-peroxidase-conjugate antibody, cat. no. sc-516102, Santa Cruz Biotechnology: https://www.scbt.com/p/m-igg-kappa-bp-hrp;
goat anti-rabbit IgG HRP antibody, cat. no. ab205718 Abcam: https://www.abcam.com/goat-rabbit-igg-hl-hrp-ab205718.html.
